# Internet skills of medical faculty and students: is there a difference?

**DOI:** 10.1186/s12909-019-1475-4

**Published:** 2019-01-30

**Authors:** Diane O’Doherty, Justan Lougheed, Ailish Hannigan, Jason Last, Marie Dromey, Colm O’Tuathaigh, Deirdre McGrath

**Affiliations:** 1 Graduate Entry Medical School, University of Limerick, Limerick, Ireland; 2 School of Medicine, University College Dublin, Dublin, Ireland; 3 School of Medicine, University College Cork, Cork, Ireland

**Keywords:** Digital, Internet skills, Medical faculty, Medical students, Medical school

## Abstract

**Background:**

The shift from a more didactic to student-centred pedagogical approach has led to the implementation of new information communication technology (ICT) innovations and curricula. Consequently, analysis of the digital competency of both faculty and students is of increasing importance. The aim of this research is to measure and compare the internet skills of medical school faculty and students and to investigate any potential skills gap between the two groups.

**Methods:**

A survey of medical school faculty and students across three universities in Ireland was carried out using a validated instrument (Internet Skills Scale) measuring five internet skills (Operational, Information Navigation, Social, Creative and Mobile). Three focus groups comprising a total of fifteen students and four semi-structured interviews with faculty across three institutions were carried out to explore further findings and perceptions towards digital literacy, give further insight and add context to the findings.

**Results:**

Seventy-eight medical faculty (response rate 45%) and 401 students (response rate 15%) responded to the survey. Mean scores for each internet skill were high (above 4 out of 5) for all skills apart from Creative (mean of 3.08 for students and 3.10 for faculty). There were no large differences between student and faculty scores across the five skills.

Qualitative results supported survey findings with a deeper investigation into topics such as online professionalism, use of licencing and mobile application development. Needs based skills training and support were highlighted as areas for faculty development.

**Conclusion:**

Both medical educators and students tend to have similar competencies with respect to internet skills. When implementing online and distance learning methodologies however, medical schools need to ensure appropriate skills training and support for faculty as well as providing targeted training to improve the creative skills of both their educators and students.

**Electronic supplementary material:**

The online version of this article (10.1186/s12909-019-1475-4) contains supplementary material, which is available to authorized users.

## Background

Medical faculty play a prominent role in educating future clinicians and advancing their skills so that their clinical work and research is informed by evidence-based practice, with the ultimate aim of improving patient care [1]. The level and nature of support given to students undertaking undergraduate and postgraduate qualifications and faculty has changed over the last few decades, as have students, faculty, curriculum and the medical education environment as a whole [2]. This has called for a change in how medical schools adapt to meet the needs of their students.

The transition from more traditional modalities of medical education (lectures, workshops and small group learning) towards online and distance learning requires educational resources of a high quality, delivered to a high standard [3]. Furthermore, while this shift in modalities offers benefits to both faculty and students [4, 5], it is dependent upon acquisition and maintenance of a certain standard of digital literacy and skills by medical faculty and students.

Although education utilizing online learning has become an increasingly useful and popular part of educational instruction, there remains some hesitance on the part of medical educators *vis à vis* use and creation of online digital content [6, 7]. Modern medical faculty are expected to have up-to-date technological skills which can be applied in devising digital content for online platforms [8]. Students are also required to be able to “hit the ground running” upon entering the workforce [9], and graduating clinicians are expected to be both familiar with different technological advancements, as well as equipped to deal with a changing healthcare environment. It is therefore essential that medical educators have the necessary digital skills and abilities in educating future clinicians [8].

To the best of our knowledge, none of the previous studies which have employed an internet skills scale has been undertaken to determine medical faculty and students’ digital skills concurrently. This suggests that there is gap in this area, particularly where a concurrent study has yet to be completed. This study therefore seeks to contribute to eliminating this gap by measuring the digital skills of medical faculty and students using a triangulation mixed methodology, employing a combination of a validated instrument [10], interviews and focus groups to facilitate a comprehensive exploration of student and medical educators’ engagement with digital content.

There are a number of questions that guide this study’s focus:

- What levels of digital internet skills do medical faculty and students report?
- Are there any gaps that might be evident between medical educators and students?
- Which socio-demographic variables influence internet skills in medical faculty and students?

## Methods

This study adopted a concurrent triangulation mixed method design, utilising quantitative and qualitative research methods, namely a large survey followed by focus groups and semi-structured interviews. Data triangulation was employed to improve confidence in the results and conclusions [11], in addition to further elaborating findings with participants’ lived experiences [12].

### Ethical approval

The study was approved by the Education and Health Science’s Ethics Committee at the University of Limerick (2016_03_01_EHS).

### Quantitative study: Internet skills scale (ISS) survey

#### Participants

Medical faculty and students from five universities were invited via independent gatekeepers (administrative staff in each university who were not connected to the research study) to participate in an online survey. The survey was distributed to both direct entry (post-secondary education entrants) and graduate entry (who had previously completed an undergraduate degree) medical students across all years of study. Only medical faculty who were responsible for both the development and delivery of the curriculum were to be invited to participate.

#### Measures

The ISS [10] has been developed and validated to measure internet skills in the general population. The ISS has a strong conceptual framework, allowing for its use in research and practice [10]. It has 35 items scored from 0 to 5 (from ‘not at all true of me’ to ‘very true of me’) and was used to measure five internet skills:

- ➢ ‘Operational’ skills scale with 10 items relates to the “skills to operate digital media”. Items on this scale include “I know how to open downloaded files”, “I know how to bookmark a website” and “I know how to adjust privacy settings”.
- ➢ ‘Information Navigation’ skills scale with 8 items relates to the ability to search for information online without navigational issues. These items were all negatively worded. Examples of items on this scale include “I find it hard to find a website I visited before” and “Sometimes I end up on websites without knowing how I got there.”
- ➢ ‘Social’ skills scale with 6 items focuses on skills for engaging in social activities on digital platforms. Items on the ‘Social’ skill scale included “I know which information I should and shouldn’t share online” and “I am careful to make my comments and behaviours appropriate to the situation”.
- ➢ The ‘Creative’ skills scale with 8 items focuses on the ability to create content. Items on this scale included “I know how to create something new from existing online images, music or video”, “I know which different types of licenses apply to online content” and “I know how to design a website”.
- ➢ The ‘Mobile’ skills scale with three items that looks at the skills necessary in using apps on mobile devices. The three items “I know how to install apps on a mobile device”, “I know how to download apps to my mobile device” and “I know how to keep tract of the costs of mobile app use”.

Demographic information was collected from students on age group, gender, country of origin, university, year of study, mode of entry to medical school (i.e. direct or graduate entry), and type of primary degree (where relevant). Demographic information from faculty included age group, gender, country of origin, university, primary degree, postgraduate qualifications (where relevant), length of teaching experience, and experience creating distance learning content.

#### Statistical analysis

Cronbach’s alpha (α) was used to measure the internal consistency of the skills scaled with values > 0.8 considered good internal consistency. Mean skill scale and item scores were calculated and compared across groups (faculty, students) using independent samples t tests. A 5% level of significance was used for all tests. Cohen’s d was used to measure effect size with 0.2 considered small, 0.5 medium and 0.8 large. Linear regression was used to predict skill scale scores using demographic data (age group, gender, country of origin) as predictors for both faculty and students. R squared was used a measure of goodness of fit of the models. Data was analysed using SPSS Statistics version 22 for Windows.

### Qualitative study: Focus groups & semi-structured interviews

#### Participants

Purposive sampling of students who expressed an interest in participating in focus groups after completing the quantitative survey was carried out, and three focus groups comprising a total of fifteen students from three medical schools were undertaken. Four semi-structured interviews also took place with medical school faculty across the three universities. Two interviews were phone interviews and two were face-to-face. In adopting a mixed method approach, results from the survey informed some of the focus group and interview questions (see Additional files 1 & 2).

#### Data analysis & synthesis

Authors reviewed all qualitative and free text data from surveys and focus group discussion to uncover similar and contrasting themes. Thematic analysis provided a framework for reviewing interview and focus group data. Authors followed Braun & Clarke’s [13] framework for thematically analysing qualitative and free-text survey data. A deductive approach was also taken to align qualitative findings with the five internet skills from ISS. Themes were coded using NVivo 10.

## Results

### Response rates

There were distributional issues with the survey in two of the five universities. In two schools, the survey was distributed to all teaching staff rather than just those involved in the development and delivery of the curriculum, which resulted in the survey being distributed to staff it wasn’t relevant for and a poor response rate. For that reason, the analysis for this study is based on three school’s data with a total response from 401 students (response rate of 15, 12% with complete data) and 78 medical faculty (response rate of 45%) across the three universities. Response rates varied across the three universities for students (7–30%) and faculty (25–57%).

### Demographics

Table 1 summarises the demographics of the students with complete responses to the survey.

**Table 1:** Demographics of student respondents (*n* = 340)^a^

Characteristic	n (%)
Gender	
Male	146 (43%)
Female	194 (57%)
Age group	
18–23	136 (40%)
24–29	174 (51%)
30–39	26 (8%)
> 40	4 (1%)
Type of entry	
Direct entry	114 (33.5%)
Graduate Entry	226 (66.5%)
Country of Origin	
Ireland	212 (63%)
Other European	15 (4%)
North American	84 (25%)
Other	29 (8%)
Year of Study	
1	73 (21.5%)
2	75 (22%)
3	83 (24%)
4	80 (23.5%)
5	10 (3%)
6	19 (6%)
Institution Response	
1	171 (30%)
2	156 (13%)
3	74 (7%)

^a^ Missing data for *n* = 61

The demographics of faculty with complete responses are summarised in Table 2. The majority (55%) were aged under 40 with less than 6 years teaching experience.

**Table 2:** Demographics of faculty respondents (*n* = 66)^a^

Characteristic	n (%)
Gender	
Male	36 (54.5%)
Female	30 (45.5%)
Age group	
< 30	23 (35%)
30–39	13 (20%)
40–49	13 (20%)
≥ 50	17 (25%)
Country of Origin	
Ireland	50 (76%)
Other European	9 (14%)
Other	7 (10%)
Teaching Experience	
< 1 Year	28 (43%)
1–5 Years	8 (12%)
6–10 Years	10 (15%)
11–15 Years	6 (9%)
> 15 Years	14 (21%)
Primary Degree	
Medicine	41 (62%)
Science	19 (29%)
Other	6 (9%)
Postgraduate Academic Qualifications	
None	25 (38%)
PhD	19 (29%)
MD	8 (12%)
MSc	4 (6%)
PG Diploma	2 (3%)
MMEd	2 (3%)
Other	6 (9%)
Institution Response	
1	11 (25%)
2	15 (37.5%)
3	52 (57%)

^a^ Missing data for *n* = 12

### Analysis of internet skills / ISS analysis

Cronbach’s alpha (α) was calculated for each skill scale (See Additional file 3). Internal consistency was good (α > 0.8) for all skill scales apart from Mobile (α = 0.63) which may reflect the small number of items on that scale (three items).

Table 3 summarises the mean skills scale scores (out of 5) by group. Mean scores were high for all skills apart from Creative. There was no statistically significant difference in mean Creative scores for faculty or students (3.10 vs. 3.08, *p* = 0.89) or mean Information Navigation scores (4.35 vs 4.23, *p* = 0.23) (Table 3). There were statistically significant differences between mean scores for Operational, Social and Mobile for students and faculty with students having higher mean scores for each skill but effect sizes were small (Cohens d ≤ 0.4, Table 3).

**Table 3:** Skills scale scores by group (Faculty, Students)

	Students (*n* = 340)	Faculty (*n* = 66)			
Subscale	Mean (SD)	Mean (SD)	Mean Difference	*p*-value	Cohen’s d
Operational	4.83 (0.32)	4.70(0.43)	0.13	0.004	0.34
Information Navigation	4.23(0.74)	4.35(0.72)	−0.12	0.23	0.16
Social	4.70(0.43)	4.49(0.74)	0.21	0.002	0.34
Creative	3.08(0.98)	3.10(1.04)	− 0.02	0.89	0.02
Mobile	4.66(0.57)	4.37(0.94)	0.29	0.001	0.37

* *p* < 0.05

Given the low mean scores for the Creative skill subscale in our study, a closer analysis was undertaken to investigate further to see which items on the subscale were poorly scored. This would allow identification of areas where support could be offered to faculty/students (See Additional file 4).

Both faculty and students scored lowest on the items “*I know which different types of licenses apply to online content”* and *“I know how to design a website”.*

### Internet skills prediction

In a multiple linear regression with socio-demographics as predictors for each skill scale, age group was a statistically significant predictor for Mobile (β = − 0.58, *p* = 0.01) and Social (β = − 0.37, *p* = 0.04) scores for medical faculty with those aged 40 or more having lower scores (Table 4). Gender was a statistically significant predictor of Mobile (β = − 0.49, p = 0.01) and Creative (β = − 3.66, *p* < 0.001) scores for medical students with female students having lower scores (Table 5). Older students (≥24 years) tended to have higher scores for Information Navigation (β = 1.33, *p* = 0.045).

**Table 4:** Linear regression of each skill scale with gender, age group and country of origin as predictors for faculty (*n* = 66)

Characteristic	Operational		Information Navigation		Social		Creative		Mobile	
	B (95% CI)	*p*-value	B (95% CI)	*p*-value	B (95% CI)	*p*-value	B (95% CI)	*p*-value	B (95% CI)	*p*-value
Gender (Female)	− 0.09 (−0.30, 0.12)	0.40	0.68 (−0.29, 0.43)	0.71	0.18 (−0.19, 0.54)	0.33	−0.17 (−0.69, 0.36)	0.52	0.12 (−0.34, 0.57)	0.61
Age group (≥ 40 years)	−0.16 (−0.37, 0.05)	0.14	0.17 (−0.18, 0.53)	0.34	−0.37 (−0.73, − 0.01)	0.04*	−0.21 (− 0.73, 0.31)	0.42	−0.58 (−1.03, − 0.12)	0.01*
Country of origin (Ireland)	−0.14 (−0.38, 0.11)	0.27	−0.14 (−0.55, 0.28)	0.52	−0.10 (−0.52, 0.32)	0.62	0.17 (−0.43, 0.78)	0.57	0.11 (−0.42, 0.63)	0.70

**Table 5:** Linear regression of each skill scale with gender, age group and country of origin as predictors for students (*n* = 340)

Characteristic	Operational		Information Navigation		Social		Creative		Mobile	
	B (95% CI)	*p*-value	B (95% CI)	*p*-value	B (95% CI)	*p*-value	B (95% CI)	*p*-value	B (95% CI)	*p*-value
Gender (Female)	−0.53 (− 1.22, 0.17)	0.14	−0.29 (− 1.57, 0.99)	0.66	0.53 (−0.04, 1.09)	0.07	−3.66 (−5.32, −1.99)	< 0.001*	− 0.49 (−0.85, − 0.12)	0.01*
Age group (≥ 24 years)	0.23 (−0.47, 0.94)	0.51	1.33 (0.03, 2.63)	0.045*	− 0.30 (−0.87, 0.27)	0.29	0.29 (−1.40, 1.97)	0.74	−0.24 (−0.62, 0.13)	0.20
Country of origin (Ireland)	−0.10 (− 0.81, 0.62)	0.79	0.46 (− 0.85, 1.78)	0.49	− 0.04 (− 0.61, 0.54)	0.91	−1.31 (− 3.02, 0.40)	0.13	0.12 (− 0.26, − 0.49)	0.54

* *p* < 0.05

### Qualitative analysis

The qualitative results provided a deeper understanding of faculty and student perceptions with respect to the five subscales of the Internet Skills Scale and any barriers that they might face when engaging with online learning. Results from the survey informed part of the questioning for the interview and focus group guides. This triangulation mixed method design was intended to provide further clarity and understanding of the topics outlined in the survey while also allowing participants to voice their opinions.

## Operational

The majority of students felt their basic operational internet skills were at a high level and were able to complete the most basic of tasks online. When asked about their confidence in their skills, experiences varied amongst faculty.

*“I think it’s probably very limited that I’m not someone that’s naturally inclined towards IT. I think it’s a personal thing.”*

Some faculty did note that they did not have a strong IT background or would not have been exposed to such technologies when gaining a medical degree via a teacher-centered traditional format but were open to expanding and improvement.

*“I would’ve come from you know fairly basic baseline. But I think that I’ve developed my skills on a need to know basis.”*

Faculty were aware that time was a constraint in learning new skills but were open to further professional development and needs based/individual training.

## Social

Interviews with faculty outlined their hesitance and resistance in using social media and the importance of online professionalism and their public profile. Not only were educators aware of their own need to remain professional, they also impressed the importance of same upon their own students.

*“I suppose it’s hugely important for the students and we’ve had this as an issue as well, your professionalism is exposed from these platforms as a doctor and as a student. So you have to be very careful that you remain… in some way censored.”*

Privacy appears to be an important determinant as to whether faculty choose to engage with social media or not.

*“I think as a clinician I want to maintain some level of privacy for me and my family. I don’t want pictures and knowledge going into the general domain.”*

Students’ use and experience of social media was very different to that of faculty. Students are using different social media, primarily to communicate class notifications, social events and resources. Students also spoke about setting up particular social groups online which allowed them to become friendly before starting their medical courses.

Issues of professional practice and confidentiality were highlighted as issues of concern for medical students in relation to social media and the sharing of information online.

*“We’ve been warned…do not put [up] any patient [identity], or any information. ‘Cos obviously in that the last few years, people have gone into hospitals…oh here’s the list for this, or here’s this interesting patient. And obviously no malice… [they] don’t really think about the patient confidentiality.”*

## Mobile

The mobile subscale was limited to three questions on the installation and download of apps on a mobile device and the cost of mobile app use hence participants did not discuss explicitly how this affected them in interviews and focus groups.

One member of faculty discussed how he along with members of the IT team, created an app which was available to medical students, helping to develop content with support from the IT team and other colleagues.

*“I’ve developed an online app with the support of the IT team…We put a lot of clinical cases on it…we have now developed a sort of a platform of digital resources.”*

## Information navigation

Faculty discussed issues of usability in relation to navigating information online, and the accessibility of different technology was also highlighted.

*“...One restriction that just came to mind was we use* (VLE name) *for our online learning materials as a repository and often times you have to click through quite a few folders to get to what you’re getting at.”*

Many students spoke about the difficulty in navigating information online, specifically relating to the lack of reliable source information for learning purposes, and they also questioned the reliability or trustworthiness of different online platforms. In the face of boundless information that could be sourced online, they noted that it was important to discern what was reputable and what was not.

*“Reliability of the source is important.”*

*“We are facing an abundance of information but sometimes they are not…well regulated.”*

Some students also expressed interest in being provided with training that would help them differentiate between reliable and reputable online resources. Whilst additional online educational sources were beneficial to students, these supplementary resources often can add to the ‘information overload’ experienced by students.

*“Sometimes I find using the internet there’s nearly information overload... there’s so much stuff…and then you can nearly be overwhelmed.”*

## Creative

Follow up interviews and focus groups allowed us to explore students’ and faculty’s experiences of creativity within medical education. The lack of knowledge on designing a website in particular was highlighted:

*“I would love to but I don’t know how to design a website or update a website.”*

Whilst most faculty were novice in their creative digital skills, one member of staff was more skilled:

*“I can grab an edit video, I can create retro graphs, I can paint pictures.”*

Members of faculty also outlined some of the concerns met when putting content online and engaging with different licenses.

*“People are reluctant. I think they are also more inclined to possibly infringe in ways that they can see. So people are happier putting up material to which they don’t have rights clearance than they are in putting up something that they feel is homemade looking”*

## Discussion

In a Web 4.0 age, it is of the utmost importance that digital skills are examined for future training opportunities and to ensure higher education institutes (including educators and students) remain competitive and innovative in technologies used in a knowledge-driven environment [14]. This study therefore sought to investigate the digital internet skills of Irish medical school students and faculty. While some differences were detected, the effect size was small, suggesting that there is no significant skills gap between faculty and students. Interestingly, both groups scored highest on the Operational skills subscale and lowest on the Creative skills subscale. Gender was a significant predictor of Mobile and Creative skill scale scores with female students scoring lower than male students. Age was also a significant predictor for Mobile and Social subscale scores for medical faculty with faculty > 40 years of age scoring lower. These findings suggest that targeted training with particular focus on creative skills may be of helpful in enhancing confidence and skills of both faculty and students in the online learning environment.

Following a deeper analysis of the low scoring Creative skills subscale, two items were identified as having been poorly answered by both groups *“I know how to design a website”* and *“I know which different types of licenses apply to online content”.* This raises the question as to the applicability of certain digital skills to medical education – do medical educators / students really need to be able to design a website? The answer is probably ‘no’. However students and faculty both scored low on the item *“I know which different types of licenses apply to online content*,” a topic which is extremely important in medical education as it is imperative that faculty and students are aware of regulations around the licensing of content online. Training of licensing digital content therefore must be considered when implementing future strategies.

Qualitative findings highlight the impact of social media in relation to a medical education setting. Social media does have a role to play in a higher education setting; the changing learning environment of higher education is supported through social media’s connectivity [15], enabling knowledge consumption and creation, and promoting “user-driven” education [16]. It is clear from this current study that students are utilising social media tools and networks such as WhatsApp and Facebook to communicate collectively, for social communications and for sharing class resources. In contrast, the majority of faculty preferred not to engage in the use of social media.

Whilst social media can foster collaborative learning and engagement [17], qualitative findings highlighted the common challenges for both faculty and students with regard to maintaining professionalism and personal and patient privacy online. Faculty members commented on the need to retain a professional profile and disengage from sharing their own lives and patient information online. This was also discussed by von Muhlen & Ohno-Machado [18] as part of their review of social media use by clinicians. The issue of patient privacy on social media networks was also been highlighted by students in this and in previous studies [19]. This hazard of “online professionalism” [19] has implications for both medical faculty and students. As argued by several authors [20, 21], students often demonstrate difficulty discerning what is appropriate and inappropriate, with an ever-growing need to maintain a professional profile online. It has been argued [22] that initiatives such as social media mentoring should be developed to ensure medical students are given guidance on how to use social media in a more professional manner, rather than advised against using it. This proactive approach appears to be a sensible solution to a rather complex issue in the twenty-first century amongst medical students, practitioners and educators.

Medical students highlighted difficulty in navigating appropriate routes of information online. The internet allows students and physicians to consume information at high speeds but the quality and reliability of this information is not always guaranteed [23]. The issue of quality control of medical information has been reviewed with possible solutions such as self-labelling by web authors and internet ‘cybermetric’ indicators [24]. It has also been argued that in order to ensure medical students are consuming the appropriate information, medical schools must provide some direction with respect to Web 2.0 resources [25]. A list of resources recommended by the medical school would therefore foster confidence in the reliability of information and at the same time promote self-directed learning. The topic of ‘information overload’ was also briefly discussed by students. While there have been tremendous advances in the quantity of medical information being made available to students online, students are often faced with too much information, resulting in time wasted, poor or delayed decision making, distraction and stress [26]. A School-approved resource list as suggested by one of the students might again help in combatting this problem.

The adoption of IT within medical education institutions is dependent on a number of variables including faculty members’ individual and institutional context in addition to their prior IT experience [27, 28]. To effectively develop innovative methods of delivering online content the value of IT must first be acknowledged by the educator [27]. Where faculty play a key role in the implementation of IT, it is important that appropriate support is made available and aligned to their various IT needs and their expectations [27].

### Strengths & limitations

To the best of the authors’ knowledge, this study is the first which utilises Van Deursen’s [10] validated instrument within a medical education setting. Our study included both faculty and medical students across all years of their medical degree programme therefore offering a distinct perspective on digital skills, supported by both statistical and thematic findings of three medical schools.

One significant limitation of this study was the poor response rate amongst faculty and students to the quantitative survey. This may be as a result of survey fatigue, both on behalf of students and faculty. The qualitative study therefore aimed to provide a deeper understanding of medical educator and students’ digital skills via data triangulation [29].

The nature of the survey as an online survey can also be seen as a limitation with delivering it in an online format possibly resulting in an over representation of those with adept internet skills. Furthermore, graduate entry students and younger faculty were over represented in our sample. This younger and more qualified population therefore may have resulted in the higher skills scores even on the Information Navigation and the Mobile subscales. Reassuringly however the findings for the other subscales were similar for the medical and general populations [10] alike.

## Conclusion

This study revealed that medical faculty and students appear to have similar competencies with respect to digital skills. However, it is essential that medical schools provide appropriate training and support for faculty in conjunction with specific creative skills, information navigation and social media training for both medical educators and students, in order to address many of the challenges faced in an expanding digital world.

## Additional files

Additional file 1: Focus Group Interview Guide. (DOCX 11 kb)

Additional file 2: Interview Guide. (DOCX 15 kb)

Additional file 3: Cronbach’s alpha for each skill (*n* = 406). (DOCX 11 kb)

Additional file 4: Creative skill items by mean score (Faculty, Students). (DOCX 12 kb)

## Abbreviations

ICT: Information communication technology
ISS: Internet skills scale
IT: Information technology
