# Supplementary material for: Internet skills of medical faculty and students: is there a difference?
Source: BMC Med Educ. 2019 Jan 30;19:39. doi: 10.1186/s12909-019-1475-4 (PMC6354327; doi:10.1186/s12909-019-1475-4)
Supplement: Supplementary file 1 — Focus Group Interview Guide. (DOCX 11 kb) [file 12909_2019_1475_MOESM1_ESM.docx]

**Additional Material 3: Focus Group Interview Guide**

1. Do you engage with the internet/digital platforms and if so what types?

2. What do you enjoy most about engaging with the internet and/or digital platforms?

3. What are your greatest concerns when engaging with the internet and/or digital platforms?

4. What barriers prevent you from engaging with the internet/digital technology?

5. What supports do you think would further improve your engagement with the internet and/or digital platforms?

6. Do you think it necessary for medical educators to be skilled in using the internet/digital platforms?

7. How might we encourage medical educators to engage better with the internet/digital platforms?
