# Supplementary material for: Internet skills of medical faculty and students: is there a difference?
Source: BMC Med Educ. 2019 Jan 30;19:39. doi: 10.1186/s12909-019-1475-4 (PMC6354327; doi:10.1186/s12909-019-1475-4)
Supplement: Supplementary file 2 — Interview Guide. (DOCX 15 kb) [file 12909_2019_1475_MOESM2_ESM.docx]

**Additional Material 4: Semi-structured Interview Guide**

1. Could you tell me a little bit about your background in regards to your education?
2. Do you engage with internet / digital platforms and if so what types?
3. Do you use any technology as part of your own teaching and or research?
4. How would you rate your level of knowledge on use of the internet and / or digital platforms?
5. Our survey results show that the social scale item questions, which look at comfort with aspects of social media, were varied amongst staff. What has your experience been with social media?
6. Results from our survey also show that creative item questions were an area which many staff felt least confident. Items in this creative scale included questions around creating new content, confidence of putting that content online and knowledge of designing a website. What has your experience been with creating digital content and putting it online?
7. How would you rate your confidence of putting your own content on a blog, website or forum?
8. How comfortable are you in using your own digital skills in your teaching and or research?
9. Do you think having / not having the necessary digital skills has added / detracted from your own teaching experience?
10. What do you enjoy most about engaging with the internet and / or digital platforms?
11. What are your greatest concerns when engaging with the internet and / or digital platforms?
12. What barriers prevent you from engaging with the internet and / or digital platforms?
13. What supports do you think would further improve your engagement with the internet and / or digital platforms?
14. Do you think it necessary for medical educators to be skilled in using the internet / digital platforms?
15. How might we encourage medical educators to engage better with the internet / digital platforms?
16. Would you attend any workshops or seminars which could help improve your digital skills, if they were provided?
17. Have you any other comments on your internet skills?
