# Supplementary material for: Internet skills of medical faculty and students: is there a difference?
Source: BMC Med Educ. 2019 Jan 30;19:39. doi: 10.1186/s12909-019-1475-4 (PMC6354327; doi:10.1186/s12909-019-1475-4)
Supplement: Supplementary file 3 — Cronbach’s alpha for each skill (n = 406). (DOCX 11 kb) [file 12909_2019_1475_MOESM3_ESM.docx]

**Supplementary Material 1:**

**Cronbach’s alpha for each skill (n=406)**

| **Skill** | **Cronbach alpha (α)** |
| --- | --- |
| Operational | 0.81 |
| Information Navigation | 0.87 |
| Social | 0.84 |
| Creative | 0.88 |
| Mobile | 0.63 |
