# Supplementary material for: Internet skills of medical faculty and students: is there a difference?
Source: BMC Med Educ. 2019 Jan 30;19:39. doi: 10.1186/s12909-019-1475-4 (PMC6354327; doi:10.1186/s12909-019-1475-4)
Supplement: Supplementary file 4 — Creative skill items by mean score (Faculty, Students). (DOCX 12 kb) [file 12909_2019_1475_MOESM4_ESM.docx]

**Supplementary Material 1**

**Creative skill items by mean score (Faculty, Students)**

| **‘Creative’ Skill Items** | **Students (n=340)** | **Faculty (n=66)** |
| --- | --- | --- |
|  | **Mean (SD)** | **Mean (SD)** |
| “I know how to create something new from existing online images, music or video” | 3.42 (1.42) | 3.48 (1.38) |
| “I know how to make basic changes to the content that others have produced” | 3.60 (1.35) | 3.86 (1.25) |
| “I know how to design a website” | 2.28 (1.42) | 2.06 (1.31) |
| “I know which different types of licenses apply to online content” | 1.83 (1.26) | 2.23 (1.33) |
| “I would feel confident in putting video content that I have created online” | 2.67 (1.43) | 2.71 (1.38) |
| “I know which apps / software are safe to download” | 3.76 (1.18) | 3.52 (1.24) |
| “I am confident about writing on a blog, website or forum” | 3.61 (1.35) | 3.47 (1.48) |
| “I would feel confident writing and commenting online” | 3.56 (1.36) | 3.48 (1.40) |
